# Disparities in Colorectal Cancer Presentation at a National Cancer Institute-Designated Cancer Center and a Safety-Net Hospital during the COVID-19 Pandemic

**DOI:** 10.18103/mra.v12i8.5761

**Published:** 2024-08

**Authors:** Munir H. Buhaya, Megan Turley, Ofelia Negrete Vasquez, Nicholas Bryant, Baqir Jafry, Sitaram Chilakamarry, Javier Salgado Pogacnik, Syed M. Kazmi, Joseph Su, Emina H. Huang

**Affiliations:** a Department of Surgery, University of Texas Southwestern, 5323 Harry Hines Blvd. Dallas, TX 75390, USA; b Department of Surgery, Parkland Health and Hospital, 5200 Harry Hines Blvd, Dallas, TX 75235, USA; c Department of Internal Medicine, University of Texas Southwestern, 5323 Harry Hines Blvd. Dallas, TX 75390, USA; d School of Public Health, University of Texas Southwestern, 5323 Harry Hines Blvd, Dallas, TX 75390, USA; e Harold C. Simmons Comprehensive Cancer Center, University of Texas Southwestern, 6202 Harry Hines Blvd, Dallas, TX 75235, USA

**Keywords:** Early onset colorectal cancer, colon cancer, rectal cancer, racial disparities, gender disparities

## Abstract

**Background:**

The clinical setting where patients with colorectal cancer (CRC), especially young adults, accessed the healthcare system during the COVID-19 pandemic to obtain their diagnosis is understudied. We hypothesized that patients with early-onset CRC (EO-CRC) present at disproportionate rates to emergency departments compared to patients with average-age onset CRC (AO-CRC).

**Patient and Methods:**

Clinical chart review was conducted for patients discussed at tumor board from the University of Texas Southwestern and Parkland Health Hospitals from August 2020 to August 2022 to compare the site of presentation that led to diagnosis: emergency department or primary care setting.

**Results:**

Two-hundred and ninety-three patients with CRC were included (69% AO-CRC, 31% EO-CRC), presenting at similar rates to primary care providers and emergency department (55% vs 45%, respectively). Most patients who presented to the emergency department received their cancer care at the safety net hospital (70%, p <0.001). Race/ethnicity, and comorbidities like obesity and metabolic dysregulation were also associated with emergency department presentation. Patients from the safety net hospital and those with obesity-related comorbidities were more likely present to the emergency department (OR 5.98, 95% CI 2.88 – 12.41, p<0.001; OR 4.18, 95% CI 1.18 – 14.81, p=0.03). Patients with rectal cancer are less likely to present to the emergency department (OR 0.42, 95% CI 0.21 – 0.85, p=0.02). No differences were observed between EO-CRC and AO-CRC with respect to the presentation site.

**Conclusion:**

Here we identified factors linked to CRC diagnostic access to the healthcare system during the COVID-19 pandemic in a racially and ethnically diverse population. Future research in this area can inform specialized CRC screening and diagnostic pathways for vulnerable young adults, guiding resource allocation to improve access to care and prompt diagnosis. Additionally, these insights can guide diagnostic access plans during global health crises for at-risk populations.

## Introduction

Colorectal cancer (CRC) is the second-leading cause of cancer death in adults in the United States of America, and it is now the leading cause of cancer mortality in men under 50 years and the second in women in this age group.^1^ CRC onset in adults younger than 50 years is defined here as early-onset colorectal cancer (EO-CRC). Despite an overall declining incidence of CRC, EO-CRC has been increasing at an alarming rate nationally and globally, becoming a global health problem.^2,3^ It is estimated that its incidence will increase to up to 124% by the end of this decade.^4^

In response to this phenomenon, the United States Preventive Services Task Force lowered the screening age recommendations for CRC in adults at average risk to 45 from 50 years in 2021, in the middle of the COVID-19 pandemic.^5^ CRC screening in the USA has remained a challenge for decades. Screening rates have been historically lower among younger adults and racial/ethnic minorities,^6–8^ and the COVID-19 pandemic further exacerbated these disparities.^9,10^ Screening rates after the updated age recommendations did not change, remaining at about 20% of the eligible adults.^11^

Suboptimal CRC screening rates have deleterious downstream effects, resulting in patients presenting with advanced disease and worse survival outcomes. Improving access to screening services in the primary care setting is one way to improve cancer outcomes. However, these opportunities are not available to patients who are ineligible to receive CRC screening. Patients younger than the screening age, now 45 years in the USA, are ineligible to access screening services, and thus they commonly present to the healthcare system after onset of symptoms. As a result, EO-CRC diagnosis is frequently delayed, resulting in higher rates of advance-stage disease at presentation and worse outcomes.^12–14^ Furthermore, this disease appears to disproportionately affect racial/ethnic minority groups who historically tend to have worse health and cancer outcomes.^15–18^ It has been shown that Black and Hispanic patients, especially those from lower socioeconomic backgrounds, have worse perioperative and oncologic outcomes relative to White patients.^18,19^ There is limited research on where young adults not eligible for CRC screening first present to the healthcare system for a diagnosis.

The COVID-19 pandemic, officially announced by the World Health Organization in Spring 2020, negatively affected cancer care. In many countries, cancer screening programs were paused, and elective procedures were postponed. For example, screening rate for colon cancer in the USA was reduced by 75%.^20^ The pandemic forced healthcare systems to shift to essential services resulting in decreased CRC screening rates, reduced incidence, delays in diagnosis, and an increase in late and emergency presentations.^21–23^ Studies examining the presentation site during the COVID-19 pandemic, which posed a significant challenge to the healthcare system and cancer care, are also scarce.

Our study aims to identify the clinical setting where patients with CRC present to the healthcare system to obtain their cancer diagnosis during the COVID-19 pandemic at a large medical center with both a safety-net tertiary care center and a National Cancer Institute-designated cancer center located in Dallas, Texas, USA. We hypothesized that patients with EO-CRC present at disproportionate rates to the emergency department (ED) versus a primary care provider site (PCP) compared to patients diagnosed with AO-CRC. We believe that our work will help us identify risk factors contributing to CRC diagnostic access inequities, potentially worsened during the healthcare strain of the COVID-19 pandemic. Our findings might help guide future research, inform specialized CRC screening and diagnostic pathways for vulnerable young adults, optimize resource allocation to enhance access to care and timely diagnosis, and prepare contingency strategies for future health crises or pandemics.

## Methods

After receiving institutional review board approval (IRB STU 2022–0279), tumor board records for a regional healthcare system (referred to as UTSW) were queried. The system shares a multidisciplinary colorectal provider team among two hospitals: a National Cancer Institute-designated cancer center (Simmons Comprehensive Cancer Center) referred to as the University Hospital (UH) and a safety-net tertiary care center (Parkland Health and Hospital System) referred as the Safety Net Hospital (SNH), both serving an urban metroplex and outlying region. Patients with new CRC diagnosis are routinely presented during this multidisciplinary tumor board. Since the UTSW medical center is a member of the National Accreditation Program for Rectal Cancer (NAPRC), all patients with rectal cancer were discussed. Records were maintained in a secure dataset. Clinical chart review of patients discussed at tumor board between August 2020-August 2022 was conducted from January 2023 to July 2023, extracting data for patient age, self-reported gender and race, comorbidities (obesity indicated by a body mass index of 30 or greater, hypertension, hyperlipidemia, and type 2 diabetes mellitus), hospital in which they received their CRC care (UH or SNH), site of malignancy (colon or rectum), disease stage at presentation, DNA mismatch repair (MMR) and *KIRSTEN-RAS* (KRAS) mutation status, initial treatment, and the clinical setting in which they accessed the health system: Emergency Department (ED) or Primary Care Provider (PCP).

Patients with hereditary cancers, a diagnosis of inflammatory bowel disease, non-adenocarcinoma, a history of colorectal adenocarcinoma presenting with recurrence, or those with incomplete data or inability to identify initial access site as ED or PCP among patients referred to our system were excluded from analysis. The final sample size was based on the number of eligible patients with complete data available for analysis. While no formal power calculation was performed, this sample size was considered sufficient to identify potential associations between the site of presentation (ED vs. PCP) and key patient demographics (e.g., age, gender, race/ethnicity). Age was defined as age at diagnosis. The prevalence of comorbidities, including hypertension, diabetes, and hyperlipidemia, was defined by the presence of these diagnosis in the patient’s problem list or prescription medication for these conditions at time of diagnosis. A metabolic dysregulation profile was assigned to patients with obesity and two additional comorbidities (hypertension, hyperlipidemia, or type 2 diabetes mellitus) or if they had all three of these comorbidities in the absence of obesity. Site of malignancy, colon or rectum, was based on endoscopic and imaging findings. Disease stage at presentation was based on American Joint Committee on Cancer staging guidelines (8th edition). The site of access to care was determined by the clinical setting in which a provider ordered the colonoscopy or imaging study by which the patient was diagnosed with CRC. For example, a PCP was identified as the access site if the patient was referred for a diagnostic colonoscopy via a traditional referral pattern. Conversely, a patient undergoing colonoscopy after having a concerning CT scan in an ED due to blood in the rectum would be considered an ED diagnosis.

### Statistical Analyses.

Fisher’s exact tests, *t* tests, Pearson’s chi-squared tests, and Kruskal-Wallis tests measured the association between EO-CRC versus AO-CRC and other variables, and the association between access site and other variables. The results of the comparative analysis, including its *p* values, were tabulated showing the total number of patients and percentages of each of the two groups per categorical variable. Logistic regression analysis was performed to determine the odds of EO-CRC compared to AO-CRC and the odds of accessing a PCP compared to the ED. All tests were performed in R (version 4.3.0, R Core Team 2023) and SPSS (version 28.0). The significance level was set at *p* < 0.05.

## Results

A total of 403 patients were discussed at the UTSW institutional tumor board from August 2020 to August 2022, and 293 patients with colon or rectal adenocarcinoma were included in our analysis. Cohort demographics by age group, AO-CRC and EO-CRC, are shown in the supplementary table. One hundred and sixty-one (55%) and 132 (45%) patients presented to a PCP and ED, respectively (Table 1). The majority of patients who presented to the ED received their cancer care at the SNH (70%), whereas most patients who presented to a PCP received their cancer at the UH (71%, p <0.001). Female patients presented to the ED compared to PCP at a higher rate (49% vs 38%), whereas male patients more frequently presented to the PCP (62% vs 51% ED, p=0.07). In our cohort, 92 (31%) patients were <50 years at diagnosis, and they presented to a PCP or the ED at similar rates (31% vs 32%, p=0.99). In examining differences by race, non-Hispanic Whites and Hispanics more frequently presented to a PCP (53%) and the ED (45%, *p*<0.001), respectively. Patients presenting to the ED had higher rates of obesity (40% vs 27% PCP, p=0.03) and metabolic dysregulation (28% vs 17% PCP, p=0.03). Patients with hypertension, diabetes, hyperlipidemia, and smoking history presented at similar rates to the ED or PCP. In examining tumor location, the proportion of patients with rectal cancer was higher in PCP (83% vs 68% ED), whereas patients with colon cancer presented at a higher rate to the ED (32% vs 17% PCP, p=0.01). Patients with advance stage disease presented a higher rate to the ED (p=0.06). No differences were observed in MMR or KRAS status.

The results of the logistic regression analysis of presentation to ED when using PCP as reference is shown on table 2. SNH patients had higher odds of presenting to the ED in both univariate (OR 5.78, 95% CI 3.49 – 9.59, p<0.001) and multivariate analysis (OR 5.98, 95% CI 2.88 – 12.41, p<0.001). Female gender also increases the odds of presenting to the ED by 59% when compared to male gender (95% CI 1 – 2.54, p=0.05). When compared to non-Hispanic White race, univariate analysis demonstrated an increase in the odds of ED presentation for Black and Hispanic patients of 248% and 283%, respectively (p<0.001). Diagnosis of obesity and metabolic dysregulation also increase the odds of presenting to the ED (OR 1.78, 95% CI 1.09 – 2.92, p=0.02; OR 1.95, 95% CI 1.1 – 3.44, p=0.02, respectively). Patients with rectal tumors were less likely to present to the ED compared to patients with colon cancer (univariate: OR 0.45, 95% CI 0.26–0.78, p= 0.004; multivariate: OR 0.42, 95% CI 0.21 – 0.85, p=0.02). Using stage 1 disease as reference, stage 3 and 4 diseases increase the odds of presenting to the ED by 257% (95% CI 1.28 – 9.99, p=0.02) and 300% (95% CI 1.36 – 11.76, p= 0.01) in univariate analysis, respectively.

## Discussion

We aimed to identify the clinical setting where patients with CRC access our regional healthcare system to obtain their cancer diagnosis during the COVID-19 pandemic. A third of the patients with CRC in our cohort were diagnosed at age 49 or younger, which is three times higher than the national average.^2,18^ This could be explained as our healthcare system is geographically located near an EO-CRC incidence hotspot.^24^ We had hypothesized that EO-CRC patients, who are not age eligible to access CRC screening services, would present to the ED with higher frequency compared to older patients. However, we did not find differences in presentation among age groups, with both groups of patients presenting at a similar rate to the ED. This could be explained by higher rates of CRC emergency and symptomatic presentation among screening-eligible adults due to non-emergent healthcare services like outpatient colonoscopies and other screening services being suspended or rescheduled during the COVID-19 pandemic.^25,26^ On the other hand, emergency colonoscopies had an increase of 2–9%.^23^

While we found no significant differences in access sites (PCP vs ED) between AO-CRC and EO-CRC, several other factors were associated with CRC presentation to the ED. Interestingly, women constituted 50% of EO-CRC patients, deviating from national trends where males typically experience a higher prevalence of EO-CRC. This observation may be attributed to the increasing rate of EO-CRC in women.^2,27^ Furthermore, the association of female gender with increased odds of ED presentation aligns with prior research that demonstrates a greater likelihood of emergency services utilization among female cancer patients.^28–31^ Additionally, women are more likely to recognize symptoms associated with CRC, prompting them to seek care earlier.^32^

Hispanic ethnicity was also associated with ED presentation. The incidence rate of EO-CRC in Hispanics has been increasing.^16,33–35^. In Texas, cancer registry data reports 26% of EO-CRC patients are Hispanic.^36^ However, in our cohort Hispanic patients represented about 40% of the EO-CRC group. We believe this is because Hispanics with EO-CRC are disproportionately represented in the UTSW catchment area.^37^ Regarding presentation to the healthcare system, Hispanics and Blacks more frequently presented to the ED, and Hispanic and Black race were associated with an increase in the odds of presentation to the ED. This finding aligns with prior research indicating higher rate of cancer diagnosis in the emergency care setting among racial minorities.^28,38^ Cancer symptom awareness has also been shown to be lower in minority and socioeconomically disadvantaged patients.^32^

In addition to gender and race/ethnic disparities, a few clinical characteristics were associated with CRC diagnosis in the ED setting. The strongest association was obesity and obesity-related comorbidities, which independently increased the likelihood of ED presentation by 4 times on multivariate analysis, aligning with findings from prior studies that shown that ED utilization is higher among those with multiple comorbidities.^29,39,40^ Comparing tumor sites, rectal cancer independently decreased the odds of presentation to the ED. Rectal bleeding is more common in rectal than colon cancer. Though symptoms at presentation was not collected, we could infer that patients with rectal cancer in our cohort were more likely to seek care from the primary provider due to more obvious symptoms like rectal bleeding and pain as opposed to more indolent generalized abdominal.^41^

Lastly, one of the strongest predictors of ED presentation was receiving cancer care at our safety net hospital (SNH). It is well established that safety-net institutions serve a large proportion of the uninsured population, and lack of insurance has been historically identified as a factor strongly associated with cancer diagnosis in the ED.^38^ Before the pandemic, only 25 % of CRC patients from UTSW’s SNH were diagnosed via presentation to a PCP.^42^ Moreover, over 80% of EO-CRC patients from UTSW’s SNH were diagnosed in the hospital setting following symptomatic presentation, with 50% of them being uninsured at the time of diagnosis.^37^

The COVID-19 pandemic brought significant challenges to CRC care, including reduced screening rates, delays in diagnosis, and higher rates of emergency presentation. Since CRC screening in the United States is suboptimal, the ED is a common site for patients to receive their CRC diagnosis, and these patients often present with late-stage disease.^43^ Even though this is less than ideal, the ED serves as a safety net for patients with heightened barriers to healthcare access. Identifying predictors of ED presentation among undiagnosed CRC patients could inform specialized screening or diagnostic pathways towards more timely diagnosis and care initiation. This is critical for young adults who are ineligible to screening services and for racial/ethnic groups disproportionately impacted by CRC. Others have already proposed to incorporate race and ethnicity as a risk factor into decision algorithm for specialized screening practices.^18^ It is important to address factors that contribute to diagnostic delays such as symptom recognition failures and healthcare access barriers in both community and healthcare settings. Prioritizing education, improving access to screening and diagnostic services, and addressing systemic barriers can significantly improve early detection and outcomes for all CRC patients, particularly those with EO-CRC and underserved populations.

This study has limitations as it is an exploratory retrospective single-institution review of patients discussed at a multidisciplinary tumor board over a 2-year period during the COVID-19 pandemic. Patients were selected from records of the UTSW’s tumor board, which introduces selection bias since patients with more advanced-stage disease and those with rectal cancer are discussed more frequently by the board. A large proportion of patients were excluded due to limited information about their presentation to the healthcare system from outside hospital records. The study includes a safety net institution, which may not reflect presentation patterns at other hospitals or regions. Patient presentation and demographics may not be representative of other multihospital healthcare systems. Hispanic patients were overly represented in this analysis, which might be reflective of the geographic location of the healthcare facilities in addition to the base population served at the UTSW’s safety net hospital. Lastly, this study reviewed patients who presented during the COVID-19 pandemic, which brings unique considerations about factors influencing utilization of healthcare services such as availability of cancer screening, access to primary care services, and emergency department visits.

## Conclusion

Our study explores an area of investigation that has been understudied in the context of the COVID-19 pandemic. Within the limitations of this study, we identified several factors that are associated with the healthcare setting where patients obtain their CRC diagnosis. This study provides a platform for further inquiry since we serve a population that is racially and ethnically diverse and is disproportionately affected by EO-CRC, which allows us to identify the factors that affect access to diagnostic and therapeutic care for patients with EO-CRC. Future studies could focus on the presentation of patients suspected of having EO-CRC to the ED, including their workup, follow-up, and outcomes. Beyond the pandemic, issues surrounding access to healthcare for red flag symptoms, often dismissed as due to benign causes such as hemorrhoids, need to be evaluated such that patients do not present in delayed fashion, and screening opportunities need to be broadened to be more inclusive. Ultimately, knowledge generated can guide resource allocation and create pathways to care for patients with EO-CRC and other vulnerable populations as well as help develop contingency strategies along the CRC care continuum during future health crises.

## Supplementary Material

1

## Figures and Tables

**Table 1.** Differences between Primary Care Provider and Emergency Department presentation among patients with colorectal cancer from the UT Southwestern Medical Center (August 2020-August 2022). AO-CRC, average-onset colorectal cancer; EO-CRC, early onset colorectal cancer; MMR, mismatch repair. Significance level: p < 0.05

	PCP (n =161; 55%)	ED (n =132; 45%)	*p* value
Hospital			<0.001
Safety net	47 (29%)	93 (70%)	
University	114 (71%)	39 (30%)	
Age Group			0.99
Less than 50 years (EO-CRC)	50 (31%)	42 (32%)	
More than 50 years (AO-CRC)	111 (69%)	90 (68%)	
Gender			0.07
Female	61 (38%)	65 (49%)	
Male	100 (62%)	67 (51%)	
Race			<0.001
Asian	17 (11%)	7 (5%)	
Black	19 (12%)	28 (21%)	
Hispanic	37 (23%)	60 (46%)	
White	85 (53%)	36 (27%)	
Undefined	3 (2%)	1 (1%)	
Comorbidities			
Obesity	44 (27%)	53 (40%)	0.03
Hypertension	64 (40%)	60 (46%)	0.39
Diabetes	35 (22%)	38 (29%)	0.21
Hyperlipidemia	54 (34%)	39 (30%)	0.55
Metabolic dysregulation	26 (16%)	36 (28%)	0.03
Smoking	63 (39%)	51 (39%)	1
Tumor location			0.01
Colon	28 (17%)	42 (32%)	
Rectum	133 (83%)	90 (68%)	
Disease stage			0.06
Stage 1	20 (12%)	5 (4%)	
Stage 2	19 (12%)	14 (11%)	
Stage 3	84 (52%)	75 (56%)	
Stage 4	38 (24%)	38 (29%)	
Molecular profile			
MMR mutation	8 (5%)	8 (6%)	0.11
KRAS mutation	22 (14%)	19 (14%)	1
Stage-concordant care	154 (96%)	126 (95%)	1

**Table 2.** Logistic regression analysis of presentation to Emergency Department when using Primary Care Provider as reference among patients with colorectal cancer from the UT Southwestern Medical Center (August 2020-August 2022). ED, emergency department; MMR, mismatch repair; PCP, primary care provider

	Univariate model			Multivariate model		
Characteristics	OR	95% CI	*p* value	OR	95% CI	*p* value
Hospital						
Safety net	5.8	(3.5 – 9.6)	<0.001	6	(2.9 – 12.4)	<0.001
University	1			1		
Age Group						
EO-CRC	1	(0.6 – 1.7)	0.89	0.9	(0.5 – 1.8)	0.82
AO-CRC	1			1		
Gender						
Female	1.6	(1 – 2.5)	0.05	1.7	(1 – 3)	0.06
Male	1			1		
Race						
White	1			1		
Asian	1	(0.4 – 2.6)	0.95	0.9	(0.29 – 2.5)	0.77
Black	3.5	(1.7 – 7)	<0.001	1.5	(0.6 – 3.7)	0.38
Hispanic	3.8	(2.2 – 6.7)	<0.001	1.3	(0.6 – 3)	0.52
Undefined	0.8	(0.1 – 7.8)	0.80	1.9	(0.2 – 21.2)	0.6
Comorbidities						
Obesity	1.8	(1.1 – 2.9)	0.02	0.9	(0.5 – 1.7)	0.74
Hypertension	1.3	(0.8 – 2)	0.33	0.9	(0.5 – 1.8)	0.80
Diabetes	1.5	(0.9 – 2.5)	0.17	0.9	(0.4 – 2.1)	0.76
Hyperlipidemia	0.8	(0.5 – 1.4)	0.47	0.37	(0.2 – 0.9)	0.02
Metabolic dysregulation	2	(1.1 – 3.4)	0.02	4.2	(1.2 – 14.8)	0.03
Smoking	1	(0.6 – 1.6)	0.93	1.3	(0.7 – 2.3)	0.47
Tumor location						
Colon	1			1		
Rectum	0.5	0.3–0.8	0.004	0.4	(0.2 – 0.9)	0.02
Disease stage						
Stage 1	1			1		
Stage 2	3	(0.9 – 9.8)	0.08	1.5	(0.4 – 6)	0.57
Stage 3	3.6	(1.3 – 10)	0.02	2.3	(0.7 – 7.4)	0.18
Stage 4	4	(1.4 – 11.8)	0.01	1.9	(0.5 – 6.7)	0.33
Molecular profile						
MMR mutation	1.2	(0.4 – 3.2)	0.75	1.5	(0.4 – 5.1)	0.57
KRAS mutation	1.1	(0.5 – 2.1)	0.87	1	(0.4 – 2.2)	0.91
Stage-concordant care	1	(0.3 – 2.9)	0.93	0.7	(0.2 – 2.7)	0.57
