# Supplementary material for: Disparities in Colorectal Cancer Presentation at a National Cancer Institute-Designated Cancer Center and a Safety-Net Hospital during the COVID-19 Pandemic
Source: Med Res Arch. Author manuscript; Available in PMC 2025 Dec 20. (PMC12716878; doi:10.18103/mra.v12i8.5761)
Supplement: 1 [file NIHMS2045953-supplement-1.pdf]

Supplementary Material

Supplementary Table

Table S1. Cohort Demographics in Patients with Colorectal Cancer from UTSW MCs' Institutional Board Review Registry (August 2020- August 2022).

	AO-CRC N=201 (69%)	EO-CRC N=92 (31%)
Gender		
Female	80 (34%)	46 (50%)
Male	121 (60%)	46 (50%)
Race		
White	91 (45%)	30 (33%)
Asian	12 (6%)	12 (13%)
Black	34 (17%)	13 (14%)
Hispanic	61 (30%)	36 (39%)
Undefined	3 (2%)	1 (1%)
Comorbidities		
Obesity	65 (32%)	32 (35%)
Hypertension	102 (51%)	22 (24%)
Diabetes	55 (27%)	18 (20%)
Hyperlipidemia	74 (37%)	19 (21%)
Metabolic Dysregulation	49 (24%)	13 (14%)
Smoking	91 (45%)	23 (25%)
Tumor Location		
Colon	48 (24%)	22 (24%)
Rectum	153 (76%)	70 (76%)
Disease Stage		
Stage 1	18 (9%)	7 (8%)
Stage 2	26 (13%)	7 (8%)
Stage 3	103 (51%)	56 (61%)
Stage 4	54 (27%)	22 (24%)
Molecular Profile		
MMR Mutation	11 (6%)	5 (5%)
KRAS Mutation	30 (15%)	11 (12%)
Hospital		
Safety Net	97 (48%)	43 (47%)
University	104 (52%)	49 (53%)

EO-CRC: Early onset colorectal cancer; AO-CRC: average onset colorectal cancer.
